# Effectiveness of a multidisciplinary care program on recovery and return to work of patients after gynaecological surgery; design of a randomized controlled trial

**DOI:** 10.1186/1472-6963-12-29

**Published:** 2012-02-01

**Authors:** Antonie Vonk Noordegraaf, Judith AF Huirne, Hans AM Brölmann, Mark H Emanuel, Paul JM van Kesteren, Gunilla Kleiverda, Jos P Lips, Alexander Mozes, Andreas L Thurkow, Willem van Mechelen, Johannes R Anema

**Affiliations:** 1 Department of Obstetrics and Gynaecology, VU University Medical Center, Amsterdam, The Netherlands; 2 EMGO Institute for Health and Care Research, VU University Medical Center, Amsterdam, The Netherlands; 3 Department of Obstetrics and Gynaecology, Spaarne Hospital, Hoofddorp, The Netherlands; 4 Department of Obstetrics and Gynaecology, Onze Lieve Vrouwe Gasthuis, Amsterdam, The Netherlands; 5 Department of Obstetrics and Gynaecology, Flevo Hospital, Almere, The Netherlands; 6 Department of Obstetrics and Gynaecology, Kennemer Gasthuis, Haarlem, The Netherlands; 7 Department of Obstetrics and Gynaecology, Amstelland Hospital, Amstelveen, The Netherlands; 8 Department of Obstetrics and Gynaecology, Sint Lucas Andreas Hospital, Amsterdam, The Netherlands; 9 Department of Public and Occupational Health, VU University Medical Center, Amsterdam, The Netherlands; 10 Research Center for Insurance Medicine, AMC-UMCG-UWV-VUmc, Amsterdam, The Netherlands

**Keywords:** Multidisciplinary care program, e-health intervention, integrated care management, participatory workplace intervention, gynaecological surgery, hysterectomy, laparoscopic adnexal surgery, return to work, work disability

## Abstract

**Background:**

Return to work after gynaecological surgery takes much longer than expected, irrespective of the level of invasiveness. In order to empower patients in recovery and return to work, a multidisciplinary care program consisting of an e-health intervention and integrated care management including participatory workplace intervention was developed.

**Methods/Design:**

We designed a randomized controlled trial to assess the effect of the multidisciplinary care program on full sustainable return to work in patients after gynaecological surgery, compared to usual clinical care. Two hundred twelve women (18-65 years old) undergoing hysterectomy and/or laparoscopic adnexal surgery on benign indication in one of the 7 participating (university) hospitals in the Netherlands are expected to take part in this study at baseline. The primary outcome measure is sick leave duration until full sustainable return to work and is measured by a monthly calendar of sickness absence during 26 weeks after surgery. Secondary outcome measures are the effect of the care program on general recovery, quality of life, pain intensity and complications, and are assessed using questionnaires at baseline, 2, 6, 12 and 26 weeks after surgery.

**Discussion:**

The discrepancy between expected physical recovery and actual return to work after gynaecological surgery contributes to the relevance of this study. There is strong evidence that long periods of sick leave can result in work disability, poorer general health and increased risk of mental health problems. We expect that this multidisciplinary care program will improve peri-operative care, contribute to a faster return to work of patients after gynaecological surgery and, as a consequence, will reduce societal costs considerably.

**Trial registration:**

Netherlands Trial Register (NTR): NTR2087

## Background

### Health care problem

Research on duration of full recovery and Return To Work (RTW) after (laparoscopic) gynaecological surgery has shown large discrepancy between expected physical recovery and actual return to work [1-3]. This may be explained by the fact that duration of recovery and RTW is mainly influenced by the expectations of the patient and employer, rather than by physical factors or the type of surgery [4-6]. However, in most cases there is hardly attention for RTW expectations [7]. In general, gynaecologists do discuss the needs and risks concerning the surgical intervention and expected duration of hospitalization with their patient, but structural convalescence recommendations regarding the resumption of (work) activities are mostly not provided [1,8]. In addition, after discharge the patient usually has only one post-operative check-up six weeks after surgery, which is focused on examination of the physical condition. Other medical care is fragmented and given only on demand, as a result of which patients often do not know whom to contact for support in case of postoperative complaints. Due to Dutch legislation, patients with paid work who do not RTW within six weeks after surgery, are generally consulted by their Occupational Physician (OP). However, as a result of the lack of recognised guidelines on the resumption of (work) activities and poor communication between the gynaecologists, General Practitioners (GPs) and OPs, often indistinct and conflicting recommendations are given and additionally most physicians do not differentiate according to the type of surgery [9-11]. These factors contribute to uncertainties and irrational beliefs of patients, which may result in delayed recovery, prolonged sick leave and reduced quality of life [12,13].

The Dutch Health Council stated, in line with the International Classification of Functioning, disability and health (ICF) model that there is a strong need for multidisciplinary recommendations for resumption of postoperative work activities [14,15]. However, little is known about patients' needs, (illness) beliefs and preferences regarding postoperative care and resumption of work activities [16]. Therefore, we previously explored patients' peri-operative needs using focus group discussions with gynaecological patients and performing a systematic review of the literature [17]. Detailed multidisciplinary convalescence recommendations were developed in collaboration with the medical board of gynaecologists, OPs and GPs through a modified Delphi consensus method with experts [18]. To mirror the target group, the focus group discussions and Delphi study were geared towards patients who underwent a hysterectomy (abdominal, vaginal, laparoscopic) or laparoscopic adnexal surgery on benign indication. These types of surgeries were chosen, because they are the most frequently performed (major) gynaecological surgical procedures with a considerable postoperative effect on recovery and RTW and yearly count for more than 17.500 procedures in the Netherlands [19].

### Multidisciplinary care program

Based on the results of the modified Delphi study, the outcomes of the focus group discussions, the literature review and considering both the ICF model as well as the Attitude, Social influence and self-Efficacy (ASE) model in which important determinants of recovery and RTW are described [20,21], a multidisciplinary care program for gynaecological patients undergoing surgery was developed. The care program aims at the different aspects of curative treatment as well as at personal and external factors. In addition, it tries to encourage patients in resuming activities and participation in the society. The program consists of an interactive e-health intervention, integrated care management and a participatory workplace intervention. The e-health intervention was developed through an intervention mapping protocol [22] and specifically aims at the empowerment of gynaecological patients and their environment during the pre- and postoperative period (from around four weeks before until eight weeks after surgery) [17]. This includes encouragement of patients in resuming daily and work activities. If the patient is still on sick leave 10 weeks after surgery, the integrated care management and participatory workplace intervention will be offered. This part of the intervention is based on a previous study with patients with chronic low back pain [23], and was adapted to our target group for this study.

### Objectives

The main objective of this study is to evaluate the effectiveness of the multidisciplinary care program (e-health intervention, integrated care management & participatory workplace intervention) compared to usual care regarding full sustainable RTW for patients after hysterectomy or laparoscopic adnexal surgery on benign indication. Secondary objectives of the study are 1) to study the effect of the multidisciplinary care program on general recovery, quality of life, pain intensity and complications; 2) to investigate how the program is evaluated by the patients, their health care providers and their employers and 3) to validate the multidisciplinary convalescence recommendations developed in het Delphi study [18].

## Methods/Design

The CONsolidated Standards Of Reporting Trials (CONSORT) statement was followed to describe the design of this study. This checklist is used worldwide to improve the reporting of Randomized Controlled Trials (RCT) [24-26].

### Organization of the study

**Figure 1:**
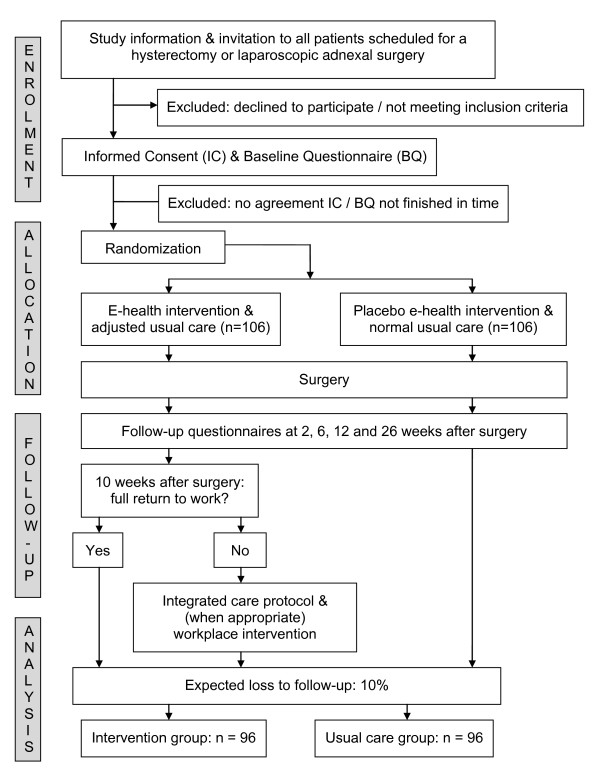
**Design of the RCT**.

The design of the study is a multicentre prospective RCT in patients undergoing gynaecological surgery and will be conducted in the Netherlands. In this study, the intervention group will receive a multidisciplinary care program (e-health intervention, integrated care management & participatory workplace intervention) and will be compared with a control group that receives usual given peri-operative care together with a placebo e-health intervention. Figure 1 presents a brief outline of the design of the study.

The seven participating hospitals in this study will be six general (teaching) hospitals and one University hospital, all localized in or nearby Amsterdam: 1)The Amstelland Hospital; 2)The Flevo Hospital; 3) The Kennemer Gasthuis; 4) The Onze Lieve Vrouwe Gasthuis; 5) The Sint Lucas Andreas Hospital; 6) The Spaarne Hospital and 7) The VU University Medical Center.

### Recruitment of patients

Participants for the study will be recruited from the waiting lists of participating hospitals. Patients scheduled for a hysterectomy or laparoscopic adnexal surgery because of benign disorders will receive an invitation letter on behalf of their gynaecologist, together with an information package consisting of: 1) patient information letter about the study, 2) Informed Consent (IC), 3) reply card to send to the researchers when not interested in participation in the study and finally, depending on the requirements of the particular hospital, 4) a leaflet about participating in scientific research in general.

When the researchers do not receive a reply card of the patient within two weeks after the delivery of the information package, they will make contact to evaluate whether she is interested in participation in the study. Phone numbers and addresses of these patients will be send to the researchers by the participating hospitals weekly. Patients willing to participate and meeting the inclusion criteria will be asked to return the signed IC and will receive the baseline questionnaire about four weeks before surgery. Subsequently, when both are filled out and the surgery is scheduled within four weeks, the patient will be randomized for the intervention or usual care group.

### Study population

Eligible patients for this study are women aged between 18-65 years, employed for at least 8 hours per week (paid or unpaid) and scheduled in one of the participating hospitals for a laparoscopic adnexal surgery and/or hysterectomy due to benign disorders. Exclusion criteria for this study are: 1) (suspicion of) malignancy; 2) (ectopic) pregnancy; 3) deep infiltrating endometriosis; 4) concomitant surgical procedures or major health problems/psychiatric disorders affecting recovery or daily activities; 5) being sick listed for more than 4 weeks, or when the surgery is to cure the reason of the absence of work, sick listed for more than 2 months; 6) working temporarily for an employment agency without detachment; 8) dealing with a lawsuit against their employer; 9) not able to understand or complete the questionnaires written in the Dutch language and 10) no access to internet.

### Randomization

To prevent unequal randomization between hospitals, patients will be pre-stratified by hospital and type of surgery (laparoscopic adnexal surgery, total laparoscopic-/laparoscopic assisted-, vaginal- and abdominal hysterectomy). A computer-generated block randomization will be performed on individual level. The blocks consist of four characters to ensure roughly equal group sizes with each stratum and are randomly varying in sequence. Randomization will be executed by an (independent) research assistant, after the patient has completed baseline measurements and IC.

### Control group; placebo e-health intervention and usual peri-operative care

In the Netherlands, there is a considerable variation in given peri-operative care and convalescence recommendations for gynaecological patients. The number of consultations and also the available time for counselling differs per hospital. After discharge, in general the patient receives one appointment for a post-operative check-up in an outpatients' department for about six weeks after surgery. Other medical care by gynaecologists and GPs is given only on demand. Patients with paid work who do not RTW within six weeks after surgery, are generally consulted by their OPs due to Dutch legislation.

In addition to given usual care, patients in the control group of this RCT will get access to a placebo e-health intervention. This website has five unique pages and provides the patient with a patient leaflet and telephone numbers of the participating hospitals. The patient leaflet is derived from The Dutch Society of Obstetrics and Gynaecology (NVOG) website and forms the basis of almost all leaflets provided in Dutch hospitals for hysterectomy or laparoscopic adnexal surgery on benign indication [27,28].

### Intervention group; multidisciplinary peri-operative care program

Patients in the intervention group, receive normal usual peri-operative care with the adjustment that the care providers supply standardized detailed convalescence recommendations to the patients [18]. In addition, this group will get access to an e-health intervention and, when they have not returned to work completely within ten weeks after surgery, supplementary care of a clinical occupational physician and (if relevant) a workplace intervention by an Occupational Therapist (OT) will be offered. This stepped care approach of additional care will be described in detail below.

#### Step 1: all patients get access to an e-health intervention from four weeks before surgery

To improve peri-operative gynaecological care, an interactive e-health intervention aiming at the empowerment of gynaecological patients during the peri-operative period including return to normal activities and work was developed [17]. This e-health intervention targets at behaviours of patients as well as of gynaecologists, GPs, OPs, employers and family members. It provides tools to compose detailed tailored instructions on the resumption of (work) activities, based on the operation date and how the surgery went (input of gynaecologist). These recommendations are based on a Delphi study among gynaecologists, GPs and OPs, using a structural consensus method including a systematic review of available literature [18]. The e-health intervention additionally provides tools (e.g. a video) to improve the communication between patients, care-providers and employers, to prevent conflicting recommendations and stimulate patients and employers to discuss potential RTW problems and to develop a work reintegration plan. Furthermore, general information on the surgical procedure itself, the (possible) consequences of the surgery and clear instructions about which symptoms require additional consultation of care providers or adaptation of convalescence recommendations, is available by the e-health intervention. In addition, it supplies an extensive list of frequently asked questions and a forum to contact other patients. Finally, patients' recovery can closely be monitored by the website, allowing the e-health intervention to advise the patient to contact a clinical occupational physician for support with reintegration when she has not RTW ten weeks after surgery. Table 1 presents an overview of the various tools of this e-health intervention.

**Table 1:** Structure of the e-health intervention [17]

Tool	Content	Involved stakeholders
1. Compose a work reintegration plan	■ Tool to compose a detailed reintegration plan with adaptations for work if necessary.	➢ Patient, Employer, OP
2. Resume normal activities	■ Tool to compose detailed advice about when normal (private) activities can be carried out again	➢ Patient, family
3. Evaluate complications	■ Estimate severity and consequences of a complication	➢ Patient, gynaecologist
4. Recovery Monitor	■ Monitoring recovery and offering assistance when relevant	➢ Patient
5. Satisfaction with recommendations	■ Evaluation and explanation of convalescence recommendations	➢ Patient
6. Satisfaction with the recovery process	■ Evaluation of satisfaction with recovery and reintegration process. Provision of advice regarding which care provider(s) to approach to receive appropriate help, when relevant.	➢ Patient
7. Invite Employer	■ Invite employer for (anonymous) section of the website which includes video (see below) and recommendations	➢ Patient, Employer
8. Video	■ Illustrate common pitfalls during the peri-operative and reintegration period	➢ Patient, employer, gynaecologist
9. Recommendations for employee	■ Advice for a successful reintegration	➢ Patient
10. Recommendations for employer	■ Advice for appropriate involvement regarding employee during the peri-operative and reintegration period	➢ Employer
11. Frequently asked questions	■ Extensive list of answers and pictures to most frequently asked questions	➢ Patient
12. Glossary	■ Explanation of most frequently used medical terms	➢ Patient
13. Forum	■ Ability to interact in public or through private messages with other patients	➢ Patient
14. Links to other websites	■ Relevant websites concerning the peri-operative and reintegration period	➢ Patient
15. Guidelines	■ Well-defined convalescence recommendations after hysterectomy and laparoscopic adnexal surgery	➢ Gynaecologists

Structure of the e-health intervention to empower gynaecological patients during the peri-operative period, including return to normal activities and work

#### Step 2: if sick leave exceeds ten weeks, additional integrated care management including workplace intervention will be offered

This part of the intervention will only be offered to the patient when she is not fully returned to work ten weeks after surgery and consists of two main protocols; 1) Integrated care protocol and 2) Workplace intervention protocol and is based on a previous study of patients with chronic low back pain [23].

##### Integrated care protocol

A clinical occupational physician will be trained as RTW coordinator to fulfil an intermediate role between the patients' gynaecologist, GP, OP and a trained OT. The clinical occupational physician independently assesses the mental and physical condition of the patient and is responsible for the planning and the coordination of the continuation of care. First consultation of the clinical occupational physician will take place in the tenth or eleventh week after surgery. Table 2 presents an overview of the integrated care protocol. Depending on the diagnosis, the clinical occupational physician will work out a treatment and rehabilitation plan (with a RTW prognosis) and discuss it with the patient and her OP. If both agree with the plan, the recommendations will be executed by calling in the assistance of the OT (if relevant), the patients' employer and/or appropriate care provider(s). The patients' OP will not lose any responsibilities regarding the final RTW plan. Communication between medical care providers will be performed according to the GP-OP-coordination guideline [29]. Six and 12 weeks after the first consultation, the patient will visit the clinical occupational physician to evaluate the progress, discuss existing problems and if necessary adjust the date of RTW.

**Table 2:** Integrated care and workplace intervention protocol

Weeks after surgery	Integrated care protocol ***Carried out by the clinical occupational physician***		Workplace intervention protocol ***Carried out by the Occupational Therapist (OT)***
**10-11**	First consultation: a. History taking and physical examination to identify: • adequacy of illness behaviour • presence of psychosocial problems • inadequate treatment • limitations at work influencing RTW (e.g. physical heavy work, organizational obstacles). b. Contact patients' other care providers (if relevant). c. Diagnosis of the medical situation or problem(s) d. Propose a treatment and rehabilitation plan (with a RTW prognosis). e. Discuss the treatment and rehabilitation plan with the patient and her occupational physician. If both agree	⤏	Contact OT to start the workplace intervention protocol (if relevant)

	↓		First consultation:
**12-13**	Call in the assistance of patients' employer and relevant care provider(s) Discuss the advisory plan (developed by the OT) with the OT	↔	a. Observation, inventory and ranking of patient's tasks and obstacles for RTW at the patients' workplace. b. Inventory and ranking patients' tasks and obstacles for RTW by the patients' employer. c. Patient, patients' employer and the OT brainstorm and discuss as many potential solutions as possible, for the problems identified in step a en b. d. Solutions are sorted and prioritized based on implementation time, costs and contribution to the problem(s). The OT reports (in consultation with the clinical occupational physician) an advisory plan specifying what has to be done, how, when and by whom. This report is sent to the patient, the patient's employer, OP and the clinical occupational physician.

**14-15**			Optional worksite visit to give additional instructions or training to the patient on working in the modified setting.

**16-17**	Second consultation: a. Evaluate the diagnosis, effect of the treatment and progress b. If necessary, adjust the date of RTW	↔	Evaluation between the patient, patients' employer and the OT (by telephone) with regard to the effects of the workplace adaptations. Further improvements are sought for when solutions have prove not to be totally effective.

**19-20**		←	Final report is sent to the patient, the patient's employer, OP and the clinical occupational physician.

**22-23**	Third and final consultation: a. Evaluate the diagnosis, effect of the treatment and progress b. If necessary, adjust the date of RTW c. Hand the employee over to her own OP		

Abbreviations: RTW = Return to Work, OT = Occupational Therapist; OP = Occupational Physician

##### Workplace intervention protocol

The workplace intervention procedure starts when the clinical occupational physician refers the patient to the OT, an expert to provide work (place) adaptations. The clinical occupational physician will define the conditions (working hours, duties, etc) under which the patient may return to work, which should be adopted by the OT and communicated effectively to the patient and the employer. The workplace intervention is based on methods used in 'participatory ergonomics' [30,31] and presumes strong commitment of both the patient and employer. This intervention has been developed originally for patients with chronic low back pain [30,32] and has shown to be (cost) effective in this population [33,34]. For this study, the protocol is adapted to post-operative gynaecological patients, regarding time-schedule and involved care providers. The consecutive steps of the workplace intervention protocol are described in table 2. The main aim of this intervention is patients' full RTW in their own or equal work. To achieve this aim, the OT will try to reach consensus between patient and her employer regarding feasible solutions for the obstacles for RTW. The solutions will be judged on short-term implementation possibilities, affordability and problem solving capability. After consensus has been reached, patient, patient's employer and OT will agree on an implementation plan, which will be evaluated during following weeks.

### Outcome Measures

#### Data-collection

The follow-up period will be 26 weeks after surgery (baseline). All the outcome variables are measured using self-report online questionnaires [35] and will be taken at baseline, 2, 6, 12 and 26 weeks after surgery. In general, the longer the recall period, the less accurate individuals are in reporting for example the use of health care services. Although no evidence on the optimal period is provided by literature, 12 and 26 weeks are frequently used and generally accepted [36]. Little agreement exists on the accuracy and validity of self-reported health care utilization and absenteeism data [37]. However, regarding sickness absence general consistency in the self-reporting can be relied on when recall is required within one month [37,38]. Therefore, in this study a monthly self-reported calendar of sickness absence per post was chosen to measure RTW. Furthermore, gynaecologists will complete questionnaires one day after surgery of each patient and at the end of the study. Employers will be asked to evaluate the e-health intervention eight weeks after the surgery of their employee. When patients, gynaecologists and employers do not fill out the questionnaires within one week, they will receive a reminder per email. If no response follows, they will be reminded by a telephone conversation. In addition to the questionnaires and calendars, the e-health intervention used by the intervention group will measure the use of the intervention, complications and (satisfaction with) recovery. Table 3 presents an overview of the outcomes and variables measured in this study.

**Table 3:** Overview of outcome measures & variables in this study

Outcome measures & variables	T_0_ Baseline^d^	T_1_ 2 wks after surgery	T_2_ 6 wks after surgery	T_3_ 12 wks after surgery	T_4_ 26 wks after surgery
Primary					

1) Return to work^a^		measured monthly^e^			

Secondary					

2) Total duration of sick leave^a^		measured monthly^e^			

3) First RTW^a^		measured monthly^e^			

4) Recovery					

• Recovery specific QoL (RI-10)	X	X	X	X	X

• Performed activities			X		

• Performed activities measured by e-health intervention^b, c^		at least 2, 4, 7, 14, 21, 28, 42, 56, 84 days after surgery			

5) Functional and general health status (SF36, EuroQoL)	X			X	X

6) Pain intensity (Von Korff)			X	X	X

7) Empowerment (GSES)		X	X	X	

8) Health care usage (TicP)				X	X

9) Occurrence of complications during the post-operative period			X	X	X

• Complications measured by e-health intervention^b, c^		at least 7, 14, 21, 28, 42, 56, 84, 126 and 182 days after surgery			

Prognostic variables					

10) Socio-demographic variables	X				

11) Type of surgery	X				

12) Complications during surgery			X		

13) Work-related factors (DMQ, JCQ, additional questions)	X				

14) Pain perception and fear avoidance belief (Tampa scale)	X				

15) Sick leave duration in the past three months	X				

16) Expectations, intention and motivation for return to work	X				

Process evaluation					

17) Patients attitudes, opinions and compliance regarding the convalescence recommendations and tools of the e-health intervention^a^		X	X	X	X

18) Physicians' and employers' attitude en opinion regarding the multidisciplinary care program^b^				X	

19) Satisfaction with care program and care providers (e.g. PSOHQ)				X	

20) Use of the e-health intervention^b^		X		X	

21) Suggestions for improvement of the e-health intervention		X		X	

22) Provided convalescence recommendations by the care providers according to the patients		X			

^a ^Return to work and total duration of sick leave is measured by a monthly calendar of sickness absence.

^b ^Only measured in the intervention group

^c ^Besides the standard requested time points, the patients can fill out the recovery monitor/complication form as many times as requested

^d ^The baseline questionnaire will be filled out no longer than four weeks before the day of surgery.

^e ^Measured from day of surgery

#### Primary Outcome measures

1) The primary outcome measure in this study is sick leave duration until full RTW, defined as: duration of sick leave in calendar days from the day of surgery until full RTW in own or other work with equal earnings, for at least four weeks without (partial or full) recurrence [39]. This means that recurrences of sickness absence within four weeks after first day of full RTW, will be considered as belonging to the preceding period of sick leave, on condition that this is due to the consequences of the surgery. RTW will be measured by a monthly calendar of sickness absence.

#### Secondary Outcome measures

2) The total duration of sick leave (due to the consequences of the surgery) during the 26 weeks follow-up period; measured by a monthly calendar of sickness absence.

3) First RTW; measured by a monthly calendar of sickness absence.

4) Recovery; measured by a validated Recovery Specific Quality Of Life questionnaire RS-QOL(RI10) [40], an extended list of (graded) activities based on the detailed convalescence recommendations given to the patients of the intervention group [18] and a recovery monitor which is a tool of the e-health intervention of the intervention group.

5) Functional and general health status (Quality of life); assessed according to the standard Dutch version of the EuroQol [41] and the Short-form health survey (SF-36) [42,43].

6) Pain intensity; measured using Von Korff questionnaire [44].

7) Empowerment; assessed by the Dutch adaptation of the General Self-Efficacy Scale [45].

8) Health care usage; measured by the Tic-P questionnaire [46] and valued according to the prices in the guidelines for economic evaluation in the Netherlands [47].

9) The occurrence of complications in the post-operative period; assessed by questions based on the complication registration form of The Dutch Society of Obstetrics and Gynaecology (NVOG) [48]. Patients in the intervention group do also answer these questions more frequently through a tool of the e-health intervention, in order to register possible side effects of the intervention and to determine and inform them when symptoms require additional consultation of care providers or adaptation of convalescence recommendations.

#### Prognostic factors

10) Socio-demographic data; measured by the standard Dutch version of the EuroQol [41] and specific additional socio-demographic questions.

11) Type of surgery.

12) Complications during surgery; assessed by questions based on the complication registration form of The Dutch Society of Obstetrics and Gynaecology (NVOG) [48].

13) Work-related factors; measured by the Dutch Musculoskeletal Questionnaire (DMQ) [49], the Job Content Questionnaire (JCQ) [50] and specific additional work-related questions.

14) Pain perception and fear avoidance belief; assessed by the Tampa scale [51].

15) Sick leave duration in the past 3 months.

16) Expectations, intention and motivation of the employee about return to work after surgery [52].

#### Outcomes regarding process evaluation

A process evaluation will be performed only in the intervention group according to the Linnan and Steckler model [53]. These patients, their gynaecologists and employers will receive specific questions regarding the multidisciplinary care program, including the e-health intervention. These questions will measure:

17) Patient's attitudes, opinions and compliance regarding the convalescence recommendations and the tools of the e-health program.

18) Physicians' and employers' attitude en opinion regarding the multidisciplinary care program.

19) Satisfaction with care program and care providers; measured with the short version of the Patient Satisfaction with Occupational Health Services Questionnaire (PSOHQ) [54] and specific additional questions related to health care and care providers.

20) The use of the e-health intervention during the follow-up period (e.g. total login time, amount of mouse-clicks, use of particular tools, et cetera).

21) Suggestions for improvement of the e-health intervention.

22) Provided convalescence recommendations by the care providers according to the patients.

### Sample size

Power calculation was performed on the primary outcome (all kind of surgeries together). To achieve a power (1-β) of 80%, with a significance level (α) of 5% and considering a HR of 1.5 in favour of the intervention group, approximately 191 patients will be needed. Anticipating a 10% drop out rate, in total a sample size of at least 212 patients will be aimed for. To recruit this number of patients, the study will anticipate on a ten-month inclusion period.

### Blinding

Patients will be blinded for the allocated treatment. Treatment allocation (randomization) will take place by computer-generated block randomization after completion of baseline questionnaire and IC. After randomization, all patients will receive access to the e-health intervention. However, after logging into the website with their personal login credentials, the kind of information provided by the e-health intervention will depend on the group the patient is randomized for.

During recruitment, the patients will be told that in case they do not RTW within 10 weeks after surgery, they might be approached for supplementary care depending on the care program they are randomized for. Nevertheless, explanation will only be given about the comparison of two different types of information supply and peri-operative guidance and not about the content of peri-operative guidance according to the 'intervention' or 'control' group.

Due to the different treatments in both groups, therapists and researchers cannot be blinded for the allocated treatment of the patient.

### Data analysis

All statistical analyses will be performed at patient level, according the Intention-To-Treat (IIT) principle. To assess whether protocol deviations have caused bias, the results of the ITT analyses will be compared to per protocol analyses in which the patients who were not treated according to the intervention protocol, will be excluded. To examine the success of the randomization, baseline characteristics of the patients in both groups will be compared using descriptive statistics. If necessary, analyses will be adjusted for prognostic dissimilarities.

A Kaplan Meier analysis (including the log rank test) will be used to describe the association between the group allocation and the duration of sick leave until the first period of full sustainable RTW. The Cox proportional hazard model will be used to estimate hazard ratios for RTW and the corresponding 95% confidence interval.

### Details of ethics approval

The study design, protocols, procedures and IC were approved by the Medical Ethics Committees of all participating hospitals: the VU University Medical Center (date 22-10-2009, number 2009/218), the Amstelland Hospital (12-02-2010, number 10-54), the Flevo Hospital (date 10-12-2009, number FZ09/35), the Kennemer Gasthuis (03-03-2010, number 2010.02), the Onze Lieve Vrouwe Gasthuis (date 21-01-2010, number 09.067), the Sint Lucas Andreas Hospital (03-11-2009, number 09/114) and the Spaarne Hospital (20-01-2010, number 561.09).

## Discussion

This paper describes a RCT to study the effect of a multidisciplinary care program on recovery and full sustainable return to work of women who underwent a hysterectomy and/or a laparoscopic adnexal surgery on benign indication. Since work participation contributes to well-being and recovery of illness, the program particularly pays attention to stimulate patients in gradually resuming normal activities including RTW [13,55]. During the first step of the multidisciplinary care program, all patients get access to the e-health intervention, which primarily aims to empower patients' behavioural determinants and supports adequate beliefs regarding recovery and RTW in patients with an uncomplicated postoperative course [18]. These are prognostic factors for recovery and RTW and account for the personal determinants in the ICF model. Secondary, the e-health intervention provides tools through which environment (e.g. family, employer), clinical condition, participation and resuming of activities may be influenced. The second step of the care program is only offered when sick leave exceeds ten weeks and thus to patients with a complicated recovery and RTW. The goal of this step is to prevent work disability. It contains additional integrated care management by a multidisciplinary team consisting of a clinical occupational physician, gynaecologist and OT and includes a workplace intervention. This step mainly focuses on reducing barriers for RTW by improving communication between different care providers, OP, employer and patient.

### Strengths and limitations

The implementation of this study in six general (teaching) and a University hospital is a good reflection of the Dutch health care situation. In addition, the selection bias of patients will be restricted through the proactive way of inviting all patients on the waiting lists to participate in the RCT. Therefore, selection will take place only based on clearly defined in- and exclusion criteria. A third strength is the blinding of the patients, which will minimize the Hawthorne and placebo effect [56]. Furthermore, the primary outcome measure in this study is full sustainable RTW, which takes into account recurrences of sick leave within four weeks after RTW and therefore reduces underestimation of work-loss days [57]. Another strength regarding the outcome measures is the evaluation of patients' health and recovery through clinical, participatory and activity outcome measures. By doing so, the influence of the care program on the different aspects of human functioning and state of health according to the ICF model, will be evaluated [14]. All patients receive their own research code according to which all data were stored in the databases. This ensures blinded analysis of the data by the researchers. Finally, this is the first study to extensively evaluate consensus-based guidelines with detailed convalescence recommendations regarding return to normal and work activities after gynaecological surgery [18,58]. The guidelines represent a consensus opinion of expert-based knowledge between gynaecologists, GPs and OPs and this study will show whether these recommendations reflect realistic recovery times for gynaecological patients and will bring about a quicker recovery without an increase in complications compared with usual care.

The main limitation of this study is that contamination between the intervention and control group cannot completely be prevented, because the randomization will be performed on patient level. With regard to the health care providers, it is impossible to blind them for the intervention allocation, because the allocation determines the kind of convalescence recommendations that should be given to the patient. Therefore, it is important for them to follow and distinguish consequently the protocol belonging to the intervention versus the control group. Contamination may occur when care providers use acquired insights received through the convalescence recommendations for the intervention group to adapt their usual care and convalescence recommendations given to the control group. To minimize this effect, we will only proactively work on the familiarity of the protocols among the health care providers and not on the detailed contents of the convalescence recommendations for the patients in the intervention group. The patients' gynaecologists will only receive a summary of the guideline by means of their patients' record, in order to pass it on to the patient at discharge from the hospital. GPs and OPs will only receive the guideline when the patient hands over her tailored convalescence recommendations or work reintegration plan to them. A learning curve of the health care providers to flawlessly execute the protocol in the intervention group may be expected. This implementation curve may result in less effect of the intervention during the beginning of the study, which should receive attention during the analysis of the results. To accelerate the implementation of the protocol, pocket maps with the description of the intervention will be distributed, teaching meetings with the health care providers about the protocol will be organized and the protocol will be added to the medical file of the patient. Although therapists and researchers cannot be blinded for the allocated treatment of the patient, they will not be involved in measuring the outcomes, since all outcome measures are self-reported and the questionnaires will be sent by email or post to the patients. Therefore, it is unlikely that the way patients complete the questionnaires, will be influenced by the researcher and care providers. Moreover, the therapists of the multidisciplinary team (RTW-coordinator, OT, and other care providers), will not be involved in the assessment of the outcomes. With concern to contamination between patients of the intervention and control group, a chance meeting cannot be excluded. According to the protocol, patients from different groups should not be placed in the same hospital room, but it cannot be prevented that patients will meet outside the room. Another limitation of this study is the fact that the integrated care management and the workplace intervention will be carried out by one clinical occupational physician and OT, which might affect the execution of the intervention. However, both will work according to a detailed standardized protocol in order to minimize their personal influence on performance of the intervention as much as possible. Compliance to e-health interventions is sometimes low. We tried to minimize the risk of low compliance as much as possible by spending extra effort and time on involving the stakeholders (end-users) in the development of this intervention and adapting it to their specific needs by use of the intervention mapping protocol [22]. Finally, although the participating hospitals reflect the proportion of (non) university hospitals in the Netherlands, they are all located in the urban agglomeration in the Western part of the Netherlands, which might be of influence on the educational level of the patient population. Because research has shown that both living in the city as well as higher educational level are associated with more frequent use of the internet for health or illness matters, it should be determined whether the educational level and internet use of the participating patients reflects that of the general Dutch population, before the results of this study can be interpreted as representative of all gynaecological patients in the Netherlands [59].

### Policy implications

Yearly more than 17.500 women receive a hysterectomy or laparoscopic adnexal surgery on benign indication in the Netherlands [19]. These large numbers of surgeries have a great impact on absenteeism since it is expected that approximately 67 percent of women aged between 25 and 65 years have paid work [60]. Therefore, if this multidisciplinary care program reduces medical consultation by providing patients with tailored, detailed and unambiguous convalescence recommendations, improves communication between care providers and stimulates patients in a faster sustainable RTW, this relatively cheap intervention may potentially decrease the sick leave costs of gynaecological patients in the Netherlands.

If the multidisciplinary guidelines evaluated in this study will bring about a quicker recovery without an increase in complications, they will be implemented broadly in the Netherlands in collaboration with the participating medical board of gynaecologists, OPs and GPs. After implementation, the expectation is that the guidelines will result in more unambiguous detailed convalescence recommendations given by gynaecologists, GPs and OPs, through which patients will be better informed about when it is medically safe to resume daily and work activities after gynaecological surgery and give them the possibility to arrange (workplace) adaptations if necessary. Furthermore, the unambiguous recommendations will likely enhance the compliance to advice given by medical specialists and stimulate the patient to resume activities with increasing gradations of strain, which will presumably bring about a quicker recovery without an increase of complications [61,62]. Therefore, the guidelines may potentially prevent work disability, increase quality of life (QoL) and increase patient satisfaction with care.

## Abbreviations

BQ: Baseline Questionnaire; GP: General Practitioner; IC: Informed Consent; ICF: International Classification of Functioning; ITT: Intention-To-Treat; OP: Occupational Physician; OT: Occupational Therapist; RCT: Randomized Controlled Trial; RTW: Return To work

## Competing interests

The authors declare that they have no competing interests.

## Authors' contributions

All authors made substantial contributions to this study and manuscript. They participated in the design of the study, the drafting and/or revising of this manuscript. All approve with this version to be published.

## Pre-publication history

The pre-publication history for this paper can be accessed here:

http://www.biomedcentral.com/1472-6963/12/29/prepub
